# Simulating Crop Evapotranspiration Response under Different Planting Scenarios by Modified SWAT Model in an Irrigation District, Northwest China

**DOI:** 10.1371/journal.pone.0139839

**Published:** 2015-10-06

**Authors:** Xin Liu, Sufen Wang, Han Xue, Vijay P. Singh

**Affiliations:** 1 Department of Water Resources and Civil Engineering, China Agricultural University, Beijing, China; 2 Department of Biological & Agricultural Engineering, Texas A & M University, College Station, Texas, United States of America; Murdoch University, AUSTRALIA

## Abstract

Modelling crop evapotranspiration (ET) response to different planting scenarios in an irrigation district plays a significant role in optimizing crop planting patterns, resolving agricultural water scarcity and facilitating the sustainable use of water resources. In this study, the SWAT model was improved by transforming the evapotranspiration module. Then, the improved model was applied in Qingyuan Irrigation District of northwest China as a case study. Land use, soil, meteorology, irrigation scheduling and crop coefficient were considered as input data, and the irrigation district was divided into subdivisions based on the DEM and local canal systems. On the basis of model calibration and verification, the improved model showed better simulation efficiency than did the original model. Therefore, the improved model was used to simulate the crop evapotranspiration response under different planting scenarios in the irrigation district. Results indicated that crop evapotranspiration decreased by 2.94% and 6.01% under the scenarios of reducing the planting proportion of spring wheat (scenario 1) and summer maize (scenario 2) by keeping the total cultivated area unchanged. However, the total net output values presented an opposite trend under different scenarios. The values decreased by 3.28% under scenario 1, while it increased by 7.79% under scenario 2, compared with the current situation. This study presents a novel method to estimate crop evapotranspiration response under different planting scenarios using the SWAT model, and makes recommendations for strategic agricultural water management planning for the rational utilization of water resources and development of local economy by studying the impact of planting scenario changes on crop evapotranspiration and output values in the irrigation district of northwest China.

## Introduction

The irrigation district in the arid area of northwest China has low precipitation and high evaporation, and runoff is not the main hydrological cycle process of this arid region. Artificial irrigation-evapotranspiration is the most important hydrological process, which forms the distinctive hydrological system in the arid irrigation district. However, because of the influence of human activities, the ecosystem there is quite fragile and a series of ecological problems have resulted, such as groundwater decline, grassland degradation, and soil desertification. Shortage of water resources has become a major obstacle to agricultural production and social and economic development in the arid region of northwest China [1]. Generally, crop evapotranspiration (ET) directly influences the hydrological cycle and the effective utilization of agricultural water resources, which plays a significant role in local food security and ecosystem conservation. Accurate measurement or estimation of crop evapotranspiration is important to develop exact irrigation scheduling and reasonably use water resources and optimize crop water management in irrigation district [2–5]. Crop evapotranspiration is also a major process influencing crop yield in water-limited environments [6, 7]. Therefore, study of crop evapotranspiration response to environmental changes is significant for developing agricultural water resources management strategies [8].

Human activities in an irrigation district are generally acknowledged as the priority factors that impact crop evapotranspiration in the irrigation district. The effect of human activities in the irrigation district mainly includes the changes of crop planting scenario, cultivation method, irrigation scheduling, fertilization and other agricultural management measures. Agricultural best management practices might contribute to sustainable water resources management in the region [9]. Different crop planting scenarios change crop evapotranspiration, soil water infiltration, canopy interception, surface runoff, etc., which all affect the water balance and hydrological processes. Thus, evapotranspiration response to land use change is one of the most obvious hydrological effects [10, 11], which plays an important role in agricultural water allocation and management in the irrigation district. Land use changes seriously influence agricultural economy, ecology and hydrology [12], and different crop types and cultivation methods will have great effects on crop evapotranspiration and water resources [13, 14]. Some factors influence crop evapotranspiration, and vary significantly in space and time for irrigation scheduling, and hence, quantification at spatial and temporal scales is of paramount importance. Hydrological model, especially the distributed hydrological model [15], is generally acknowledged to be one of the most effective methods to study evapotranspiration response to land use change. A physically-based distributed model is preferable, since it can realistically represent the spatial variability of catchment characteristics [16]. Because of the distributed nature, the model can be used to simulate regional spatial changes of key hydrological process components. Different land cover scenarios can be set according to the local development policy or simulated by a land use change simulation model, such as CLUE-S model [17, 18]. Then, the output data of different land cover scenarios can be used as input data for regional evapotranspiration simulation in the hydrological model. Other method to study the influence of land use change on evapotranspiration includes combining remote sensing with a surface energy balance algorithm for land and evapotranspiration modelling [19–21].

Recently, the Soil and Water Assessment Tool (SWAT) model has been widely used for simulation of the water movement process response to environmental change to combat water and ecological problems because of the advantages the models has, such as multiple functions, modular design and opening program code [22, 23]. Numerous studies have used SWAT to evaluate the impact of agricultural water management practices on water resources [9]. However, the SWAT model is developed for hydrological processes simulation on a basin scale. The interaction between surface water and groundwater, and the frequent exchange of unsaturated zone soil water and groundwater are simplified [24]. Also, there are limitations to apply the model to irrigation districts, and the applicability of this model over different geographic characteristics areas is still an open question. Therefore, many investigators have focused on research into hydrological processes in irrigation areas by incorporating improved modules for different purposes [25–27]. Few studies employ the essential adaptations of crop evapotranspiration for local soil and meteorological conditions in evapotranspiration-dominant arid areas using the SWAT model. Hence, crop evapotranspiration response to different planting scenarios needs to be investigated in arid areas.

The objectives of this study therefore are (1) to improve SWAT model by transforming the evapotranspiration module, (2) to apply the original and improved model to a case study in an irrigation district of arid area of Northwest China for model calibration and verification, and (3) to simulate crop evapotranspiration response to different planting scenarios.

## Materials and Methods

### 2.1 SWAT model

The study simulates evapotranspiration using the SWAT model. The Soil and Water Assessment Tool (SWAT) has been developed by the USDA Agricultural Research Service [24]. SWAT is a distributed process-based hydrological model that can operate on a daily or monthly time step. It allows for a number of different physical processes to be simulated in a basin. Hydrological Response Units (HRUs) are the basic calculating units, which consist of unique combinations of land resources and soil types in each sub-basin. The simulation processes of SWAT include major components, such as hydrology, meteorology, soil, land use, crop structure, and agricultural management [28]. The water balance equation in the SWAT model is expressed as

$$SW_{t}=SW_{0}+\sum_{i=1}^{t}(R_{day}+Q_{surf}+E_{\alpha }-\omega_{seep}-Q_{gw})$$ (1)

where *SW*_*t*_ is the final soil water content (mm), *SW*_0_ is the initial soil water content (mm), *t* is the time (d), *R*_*day*_ is the amount of precipitation on day *i* (mm), *Q*_*surf*_ is the amount of surface runoff on day *i* (mm), *E*_*α*_ is the amount of evapotranspiration on day *i* (mm), *ω*_*seep*_ is the amount of percolation that bypasses the soil profile bottom on day *i* (mm), *Q*_*gw*_ is the amount of return flow on day *i* (mm).

### 2.2 Original evapotranspiration module in SWAT

In SWAT model, the potential and actual evapotranspiration will be calculated first, and then the relatively smaller value will be chose as the regionally actual evapotranspiration.

#### 2.2.1 Potential evapotranspiration

The methods of Penman-Monteith, Priestley-Taylor and Hargreaves are used for crop evapotranspiration calculation in SWAT model. Besides, the measured data can also be used as the potential evapotranspiration data.

#### 2.2.2 Actual evapotranspiration

The actual evapotranspiration include canopy interception, crop transpiration and soil evaporation in SWAT model.

(1) Canopy interception

If the potential evapotranspiration is less than the free moisture intercepted by the canopy, then,

$$E_{a}=E_{can}=E_{0}$$ (2)

$$E_{can}=E_{INT(i)}-E_{INT(f)}$$ (3)

where *E*_*a*_ is the actual evapotranspiration (mm), *E*_*can*_ is the evapotranspiration of free moisture of canopy (mm), *E*_0_ is the potential evapotranspiration (mm), *E*_*INT*(*i*)_ is the initial free moisture content of canopy (mm), and *E*_*INT*(*f*)_ is the finial free moisture content of canopy (mm).

If the potential evapotranspiration is more than the free moisture intercepted by the canopy, then,

$$E_{can}=E_{INT(i)}$$ (4)

$$E_{INT(f)}=0$$ (5)

When the free moisture intercepted by the canopy has been evaporated, then, the moisture from crop and soil will be evapotranspired.

(2) Crop transpiration

The crop transpiration is calculated as:

$$E_{0}^{\prime }=E_{0}-E_{can}$$ (6)

$$\begin{array}{l} E_{t}=\frac{E_{0}^{\prime }\cdot LAI}{3.0}\;0\leq LAI\leq 3.0\\ E_{t}=E_{0}^{\prime }\;LAI>3.0 \end{array}$$ (7)

where $E_{0}^{\prime }$ is the potential evapotranspiration after the free moisture of canopy has been evaporated (mm), *E*_*t*_ is the maximum transpiration (mm), and *LAI* is the leaf area index.

(3) Soil evaporation

The soil evaporation depends on the soil depth, and it can be calculated as:

$$E_{soil,\;z}=E_{s}^{\prime \prime }\cdot \frac{z}{z+exp\left(2.347-0.00713\times z\right)}$$ (8)

where *E*_*soil*, *z*_ is the moisture that can be evaporated at the depth of *z* (mm), $E_{s}^{\prime \prime }$ is the allowed maximum evaporation (mm), and *z* is the soil depth below the ground surface. The function of coefficients in the equation is to make sure that 50% of the evaporation is from the soil depth of 0–10mm and 95% of the evaporation is from the soil depth of 0–100mm.

The moisture that can be evaporated at the soil layer depends on the moisture that can be evaporated at the upper and lower layers of the soil.

$$E_{soil,\;ly}=E_{soil,\;zl}-E_{soil,\;zu}$$ (9)

where *E*_*soil*, *ly*_ is the moisture that can be evaporated at the soil layer of *ly* (mm), *E*_*soil*, *zl*_ is the moisture that can be evaporated at the lower layer of the soil (mm), and *E*_*soil*, *zu*_ is the moisture that can be evaporated at the upper layer of the soil (mm).

### 2.3 Development of evapotranspiration module

In an arid and semi-arid irrigation district, irrigation-evapotranspiration is the main hydrological process, and crop transpiration and soil evaporation are the main water consumption factors. Improving the evapotranspiration module can be an effective approach to simulate evapotranspiration in the arid and semi-arid irrigation district. As FAO–56 dual crop coefficient can accurately predict evapotranspiration, transpiration and evaporation for different crop types under different mulching, and can also estimate short time step values, it is more suitable for evapotranspiration simulation of different crops [4]. Thus, the method was introduced into the SWAT model to improve the evapotranspiration module. The FAO–56 dual crop coefficient method can be calculated as [29]:

$$ET_{c}=k_{c}ET_{0}$$ (10)

$$k_{c}=(k_{cb}+k_{e})\;ET_{0}$$ (11)

where *ET*_*c*_ is the crop evapotranspiration (mm), *k*_*c*_ is the crop coefficient, *ET*_0_ is the reference crop evapotranspiration, *k*_*cb*_ is the basal crop coefficient, and *k*_*e*_ is the soil evaporation coefficient.

#### 2.3.1 Basal crop coefficient

The basal crop coefficient, *k*_*cb*_, is obtained from FAO–56 tabulated recommendations, incorporating the effect of crop and climate factors:

$$k_{cb}=k_{cb(Tab)}+\left[0.04\left(u_{2}-2\right)-0.004\left(RH_{min}-45\right)\right]{\left(\frac{h}{3}\right)}^{0.3}$$ (12)

where *k*_*cb*(*Tab*)_ is the basal crop coefficient value of different growth stage in the FAO–56 table, *u*_2_ is the wind speed at 2.0 m height (m/s), *RH*_*min*_ is the minimum daily relative humidity (%), and *h* is the crop height (m).

#### 2.3.2 Soil evaporation coefficient

The soil evaporation coefficient, *k*_*e*_, is calculated by the FAO–56 recommendations method:

$$k_{e}=k_{r}\;(k_{c\;max}-k_{cb})$$ (13)

where *k*_*r*_ is the soil evaporation reduction coefficient and is related to the cumulative depth of water depleted from the topsoil layer, and *k*_*c max*_ is the maximum value of *k*_*c*_ following precipitation and irrigation:

$$k_{c\;max}=max\left(\left\{1.2+\left[0.04\left(u_{2}-2\right)-0.004\left(RH_{min}-45\right)\right]{\left(\frac{h}{3}\right)}^{0.3}\right\},\;\left\{k_{cb}+0.05\right\}\right)$$ (14)

The calculation of soil evaporation reduction coefficient is divided into two stages by the FAO–56 procedure: energy limiting stage and soil water supply capacity controlling stage. In the first stage, the soil water is adequate and evaporation is restricted by energy, so *k*_*r*_ = 1. In the second stage, when *D*_*e*,*i*−1_ > *REW*, the evaporation starts dampening, and the soil evaporation reduction coefficient is calculated as

$$k_{r}=\frac{TEW-D_{e,i-1}}{TEW-REW}$$ (15)

where *REW* is the cumulative depth of evaporation by the end of the energy limiting stage (mm), *TEW* is the total evaporable water (mm), and *D*_*e*, *i*−1_ is the cumulative depth of evaporation from the soil surface layer by day *i*–1 (mm).

As the parameters are hard to obtain in the second stage, the soil evaporation reduction coefficient can be calculated as

$$k_{r}=\frac{FC-S_{t}}{FC-0.5WP}$$ (16)

where *FC* is the field capacity (mm), *S*_*t*_ is the soil water content (mm), and *WP* is the wilting coefficient (mm).

### 2.4 Framework of the study

**Fig 1:**
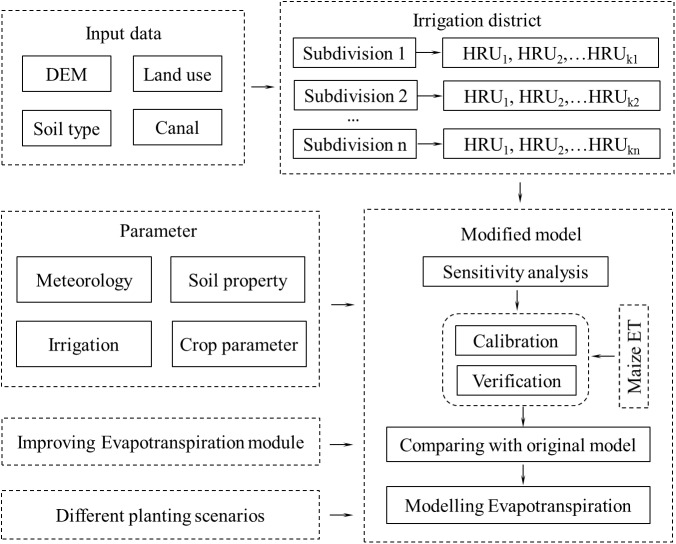
Framework of the study.

In this study, the data of DEM, land use, soil type and canal will be first put into the SWAT model for the irrigation district subdivision. Then, the evapotranspiration module of the model will be modified, and the parameters of meteorology, soil property, irrigation and crop will also be put into the modified model for the sensitivity analysis of parameters that are related to crop evapotranspiration. Thereafter, the summer maize evapotranspiration will be used for model calibration and verification, and results will also be compared with the original SWAT model to verify that the modified model is more suitable for simulating crop evapotranspiration in the irrigation district of northwest China. Finally, the crop evapotranspiration response to different planting scenarios will be simulated in order to put forward the suitable planting scenario according to the local policy. The framework of the study is presented in Fig 1.

### 2.5 Ethics Statement

Wuwei and Qingyuan Water Resources Administrative Bureau granted permission for modelling crop evapotranspiration response to different planting scenarios in Qingyuan Irrigation District of Shiyang River basin, the arid region of northwest China.

## Case Study

### 3.1 Study area

**Fig 2:**
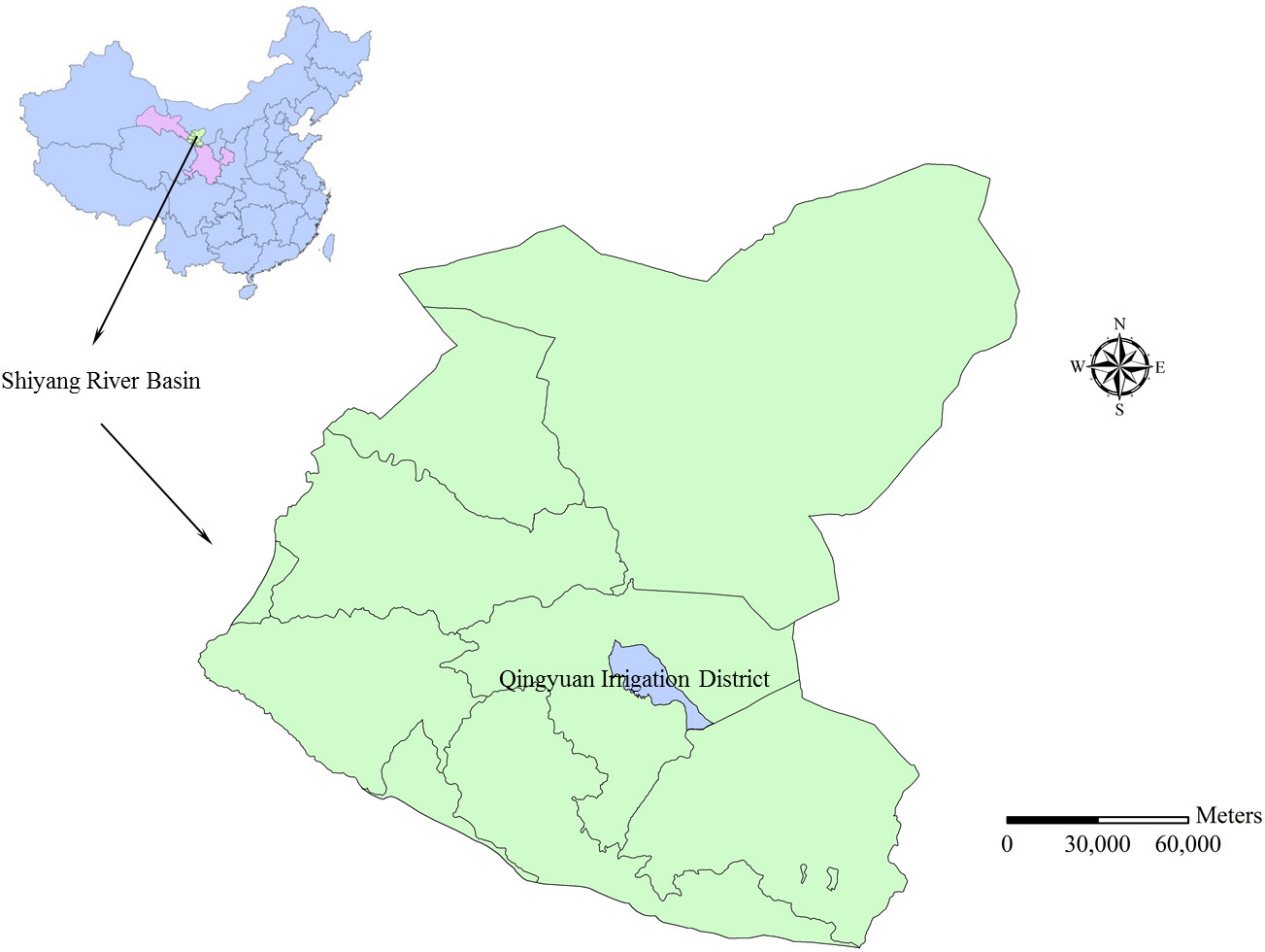
Location of Qingyuan Irrigation district.

**Fig 3:**
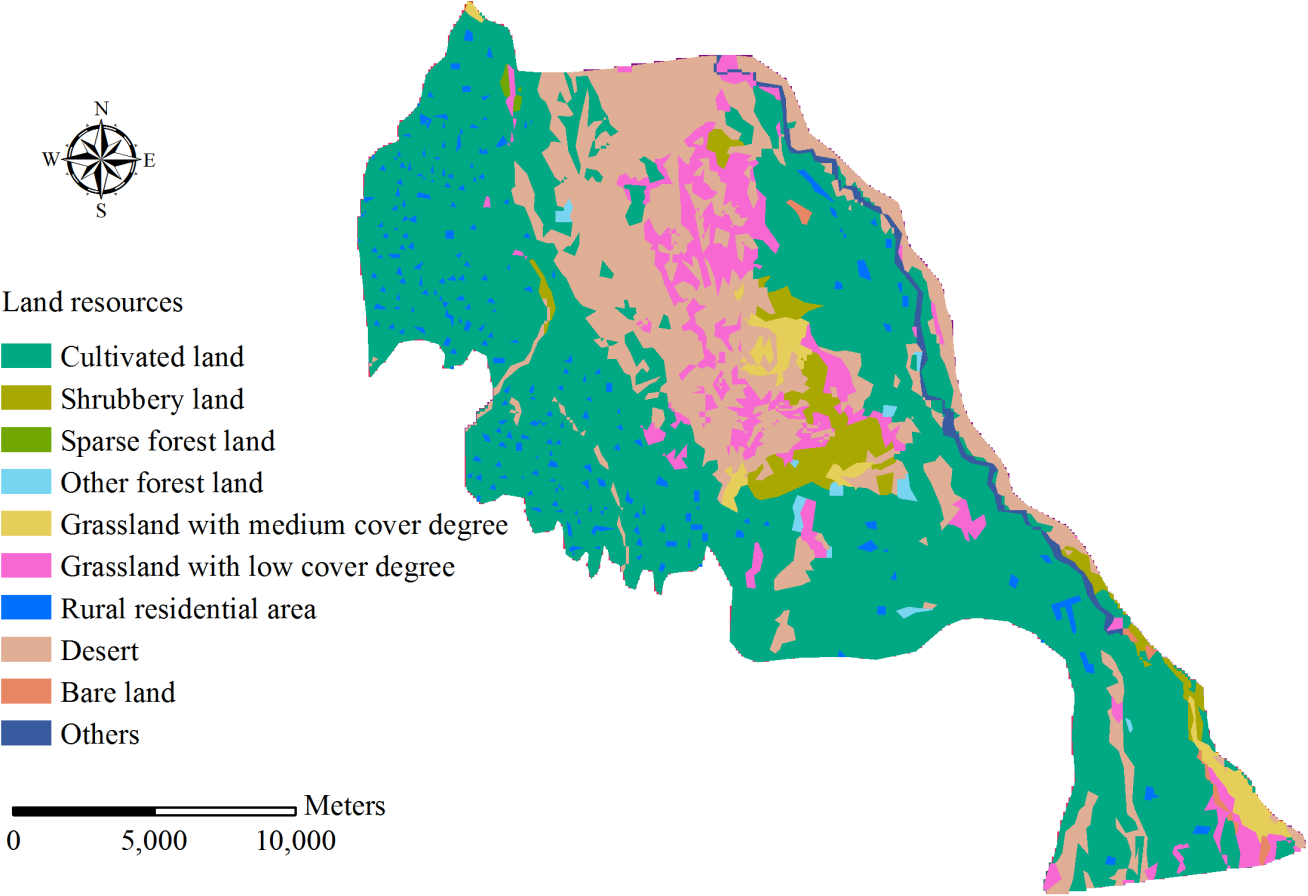
Land resources in Qingyuan Irrigation District.

**Fig 4:**
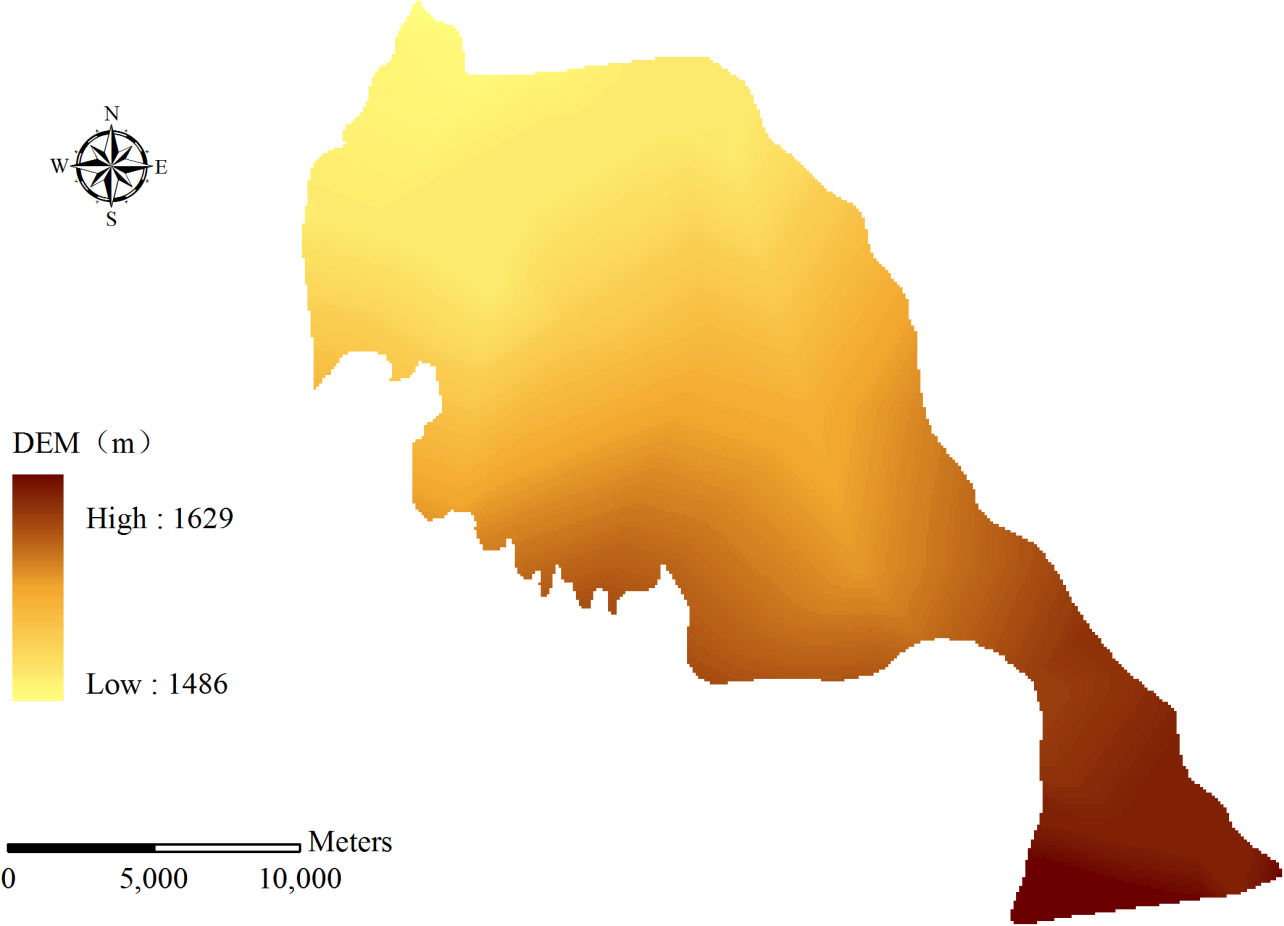
DEM of Qingyuan Irrigation District.

**Fig 5:**
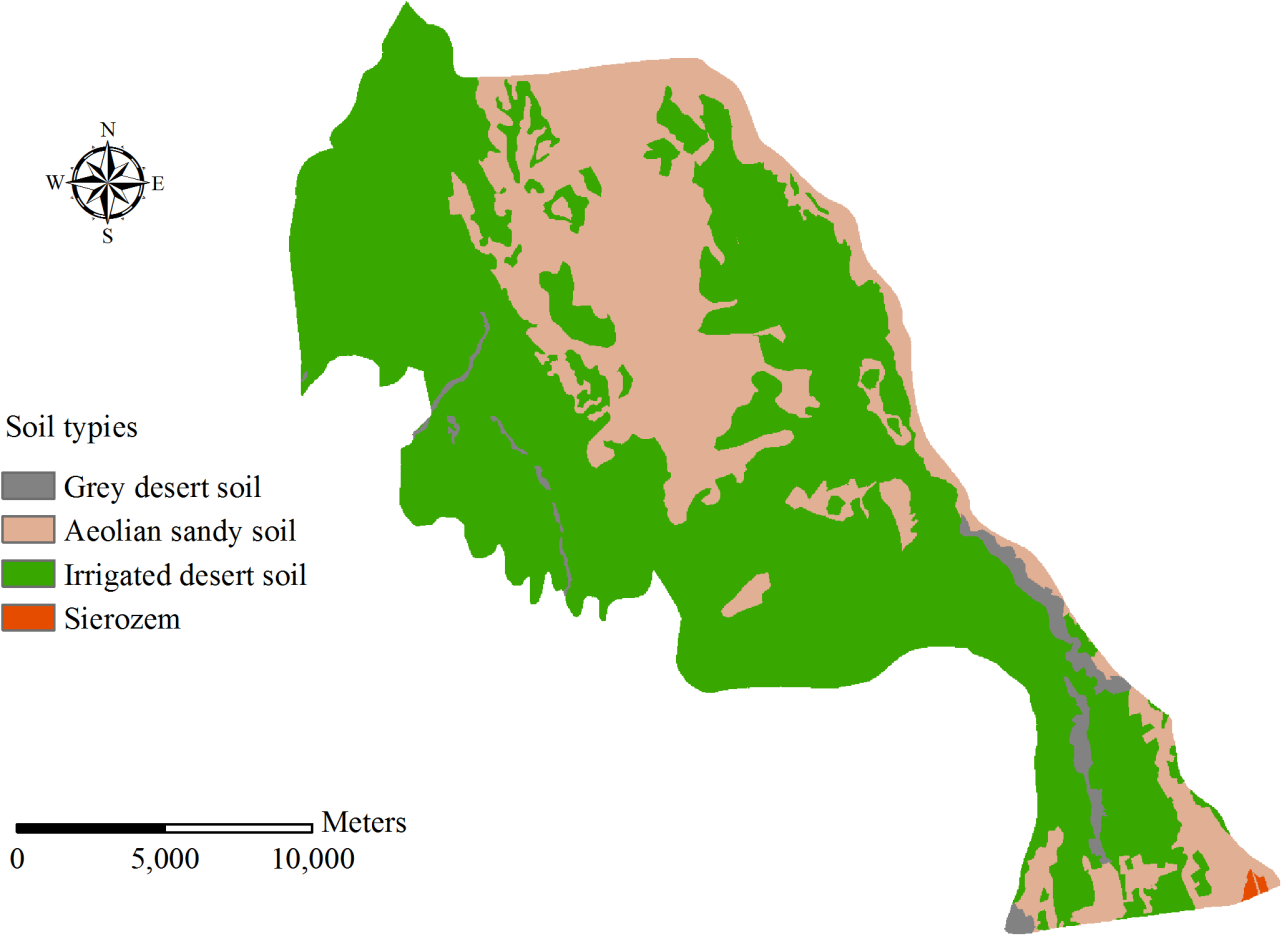
Soil types in Qingyuan Irrigation.

Qingyuan Irrigation District is located in the middle reach of Shiyang River basin, the arid region of northwest China. The total area of the irrigation district is approximately 390 km^2^ (Fig 2). The climate of the area is temperate continental climate. The annual precipitation is 150–160 mm, and precipitation occupies 80–85% from April to September. Annual evaporation is 2020 mm and average temperature is 7.7°C. The land resources in Qingyuan Irrigation District can be divided into cultivated land, forestland, grassland, rural residential area, unused land and others (Fig 3). Cultivated land constitutes 59.99% of the total land resources and the proportions of other land types are presented as Table 1. The terrain inclines downward from southwest to northeast in the irrigation district. The longitudinal slope is 1/800–1/200 and the altitude is 1500–1600 m. The digital elevation model (DEM) of Qingyuan Irrigation District is shown in Fig 4. There are four soil types in the irrigation district: grey desert soil, irrigated desert soil, aeolian sandy soil and sierozem (Fig 5). Spring wheat and summer maize are the main grain crops, and potato, soybean, beet and chili are the main economic crops in Qingyuan Irrigation District.

**Table 1:** Proportions of different land resources in Qingyuan Irrigation District.

Land resources		Proportion (%)
Cultivated land	Cultivated land	59.99
Forestland	Shrubbery land	3.50
	Sparse forest land	0.11
	Other forest land	0.51
Grassland	Grassland with medium cover degree	1.98
	Grassland with low cover degree	7.41
Rural residential area	Rural residential area	2.51
Unused land	Desert	22.45
	Bare land	0.33
Others	Others	1.22

### 3.2 Input data

#### 3.2.1 DEM

In order to simulate crop evapotranspiration in irrigation district, the DEM of study area should be put into the model. For Qingyuan Irrigation District, the topographical data to form the DEM was obtained from 1:250000 resolution maps (Fig 4).

#### 3.2.2 Land use database

Land use data of Qingyuan Irrigation District were obtained from a 100 m×100 m resolution map (Fig 3), and the main land use types are cultivated land, forestland, grassland, rural residential area, unused land, and others.

#### 3.2.3 Soil database

Soil type data were obtained from a 100 m×100 m resolution map. The soil physical properties include bulk density, available water capacity, saturated hydraulic conductivity and soil particle composition (Table 2). Soil properties of different types were measured by collecting different soil types from different soil layer depths distributed in typical areas of Qingyuan Irrigation District. The soil bulk density and available water capacity were measured by the Wilcox method. The saturated hydraulic conductivity was measured by the constant head penetration test. Soil particle composition was measured by Malvern particle size analyzer (MS 2000). The soil properties classification criterion is based on the USDA classification system, which is the same as the criterion in the SWAT model.

**Table 2:** Soil physical properties in Qingyuan Irrigation District.

Soil types	Soil depth (mm)	Bulk density (g/cm^3^)	Available water capacity	Saturated hydraulic conductivity (mm/h)	Soil particle composition		
					Clay content (%)	Silt content (%)	Sand content (%)
Grey desert soil	300	1.59	0.14	33.41	6.43	19.78	73.80
	600	1.40	0.17	32.00	11.94	40.31	47.75
Irrigated desert soil	200	1.52	0.17	12.64	16.05	46.48	37.47
	400	1.40	0.12	11.94	21.97	48.28	29.75
	600	1.46	0.07	8.13	25.71	68.03	6.26
	800	1.51	0.09	11.43	19.24	69.54	11.22
	1000	1.53	0.07	8.38	26.74	62.17	11.08
Aeolian sandy soil	500	1.40	0.05	151.38	1.14	5.71	93.14
	1000	1.40	0.05	120.65	3.10	13.99	82.91
Sierozem	210	1.20	0.14	2.18	21.00	36.00	43.00
	450	1.20	0.14	3.73	14.00	43.00	43.00
	1070	1.40	0.14	3.57	16.00	51.00	33.00
	1500	1.40	0.22	3.26	12.00	75.00	33.00

#### 3.2.4 Meteorological database

The daily meteorological data (daily precipitation, maximum and minimum temperatures, wind speed, average and minimum relative humidity) at Wuwei Meteorological Station from 1960 to 2011 were selected for hydrological simulation.

#### 3.2.5 Irrigation scheduling

Irrigation scheduling has a great impact on crop evapotranspiration, so it is an important element for evapotranspiration simulation in the irrigation district. Table 3 presents the irrigation time and irrigation quota of main crops in Qingyuan Irrigation District. The irrigation scheduling is under conditions of border irrigation and furrow irrigation.

**Table 3:** Irrigation scheduling in Qingyuan Irrigation District.

Crop	Irrigation time	Irrigation quota (mm)
Spring wheat	May 6th	105
	May 26th	105
	June 13th	105
	July 6th	97.5
Summer maize	June 13th	105
	July 6th	112.5
	August 4th	112.5
	August 24th	105
Potato	May 27th	97.5
	June 16th	97.5
	July 7th	97.5
	July 30th	90
Soybean	May 25th	90
	June 15th	97.5
	July 6th	105
	August 4th	105
Beet	May 15th	97.5
	June 4th	105
	July 1st	105
	August 6th	105
Chili	May 20th	97.5
	June 15th	97.5
	July 1st	97.5
	July 28th	97.5
Watermelon	May 9th	75
	May 27th	82.5
	June 16th	75
	July 7th	75
	July 26th	67.5

#### 3.2.6 Crop coefficient

As the evapotranspiration module has been modified, the basal crop coefficient cannot be calculated in the SWAT model directly. Table 4 presents the basal crop coefficient for different growth stages of main crops.

**Table 4:** Basal crop coefficient values of different crops in Qingyuan Irrigation District.

Crop	Basal crop coefficient		
	*k*_*cini*_	*k*_*cmid*_	*k*_*cend*_
Spring wheat	0.30	1.27	0.82
Summer maize	0.30	1.19	0.80
Potato	0.15	1.10	0.65
Soybean	0.15	1.10	0.30
Beet	0.15	1.10	0.95
Chili	0.15	1.10	0.80
Watermelon	0.15	1.05	0.75

### 3.3 Subdivision of irrigation district

**Fig 6:**
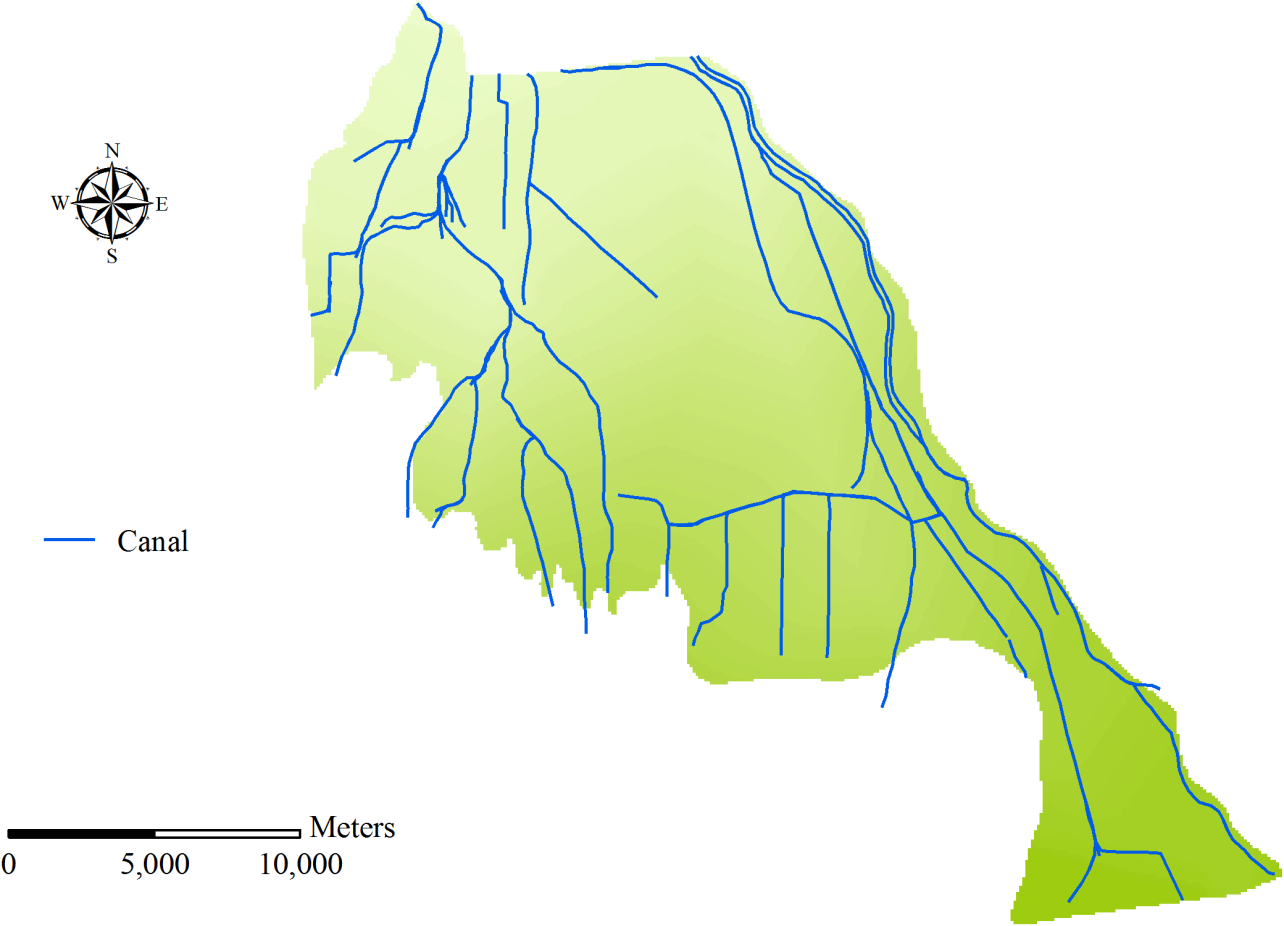
Distribution of canal system in Qingyuan Irrigation District.

**Fig 7:**
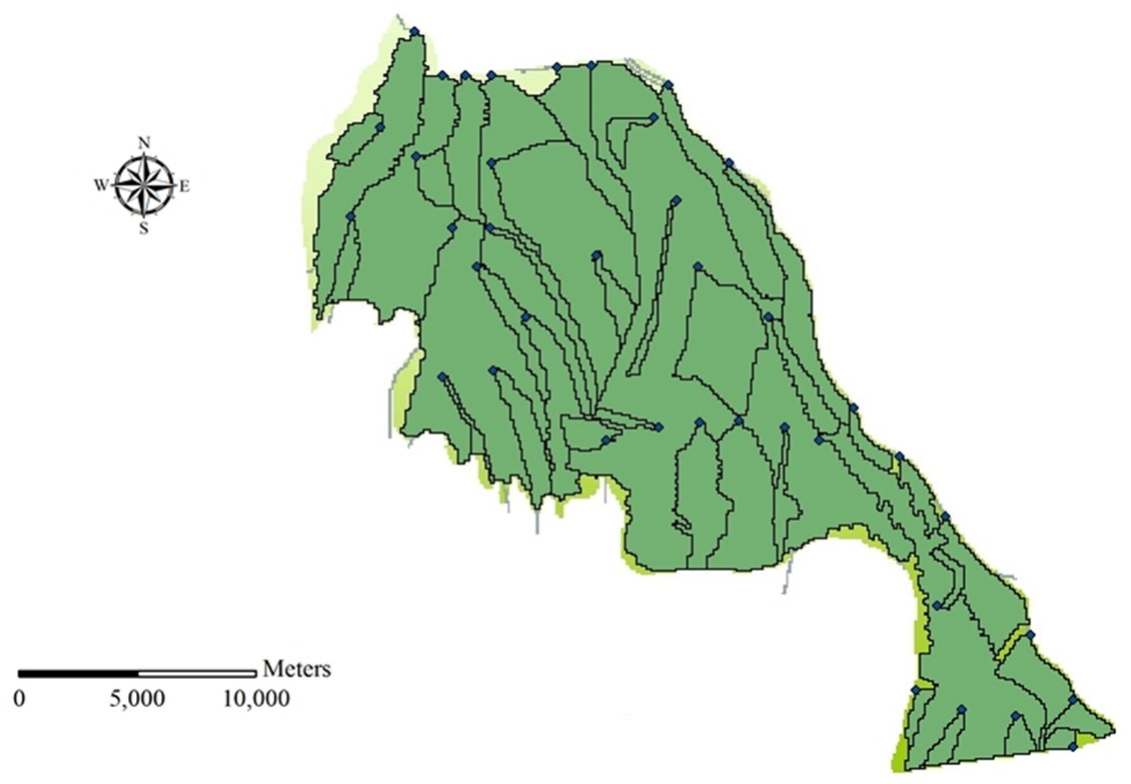
Subdivision of Qingyuan Irrigation District.

Generally, catchment can be divided into a number of sub-catchments based on terrain characteristics in the SWAT model. However, the area of Qingyuan Irrigation District is small. The terrain inclines downward from southwest to northeast and there is no watershed in the irrigation district. Besides, the water systems are artificial canal systems which have a great impact on the natural hydrological processes. Therefore, the irrigation district which is divided based on terrain characteristics by the function of SWAT cannot reflect the real situation of the study area. As irrigation system is the main factor that influences hydrological cycle, the irrigation district was divided mainly based on the canal systems. Then, taking into account the factors of DEM, land use and soil type, the whole irrigation district was divided into 39 subdivisions and 145 HRUs. The distribution of canal system and subdivision of Qingyuan Irrigation District is shown as Fig 6 and Fig 7.

### 3.4 Sensitivity analysis

Sensitivity analysis is important for distributed hydrological model simulation with numerous parameters. The LH-OAT analysis method [30] was used in this study. The parameters (soil evaporation compensation factor, maximum canopy interception, shallow groundwater re-evaporation coefficient, plant absorb compensation factor and soil available water) which have influences on crop evapotranspiration were used for sensitivity analysis based on monthly measured values of summer maize evapotranspiration and model simulations for the period of 1990–2000. The parameter ranges used in model sensitivity analysis are presented in Table 5.

**Table 5:** The results of sensitivity analysis for the SWAT parameters for summer maize evapotranspiration in Qingyuan Irrigation District.

Parameter	Definition	Rank	Parameter range	Parameter calibration value	Process
v_ESCO.hru	Soil evaporation compensation factor	1	0.0–1.0	0.875	Evaporation
v_CANMX.hru	Maximum canopy interception	2	0.0–30.0	9.853	Evaporation
v_GW_REVAP.gw	Shallow groundwater re-evaporation coefficient	3	0.02–0.2	0.115	groundwater
v_EPCO.hru	Plant absorb compensation factor	4	0.0–1.0	0.827	Evaporation
r_SOL_AWC(1).sol	Soil available water	5	-0.5–0.5	0	Soil

### 3.5 Model calibration and verification

Summer maize is one of the main crops in the study area and its growing period is from May to September when the evapotranspiration is relatively higher compared with months. Thus, the measured values of summer maize evapotranspiration were used for model calibration and verification. The measured values are from the experimental data of Shiyanghe Experimental Station for Water-saving in Agriculture and Ecology. The data were divided into two parts: a calibration period (1990–2000) and a verification period (2006–2011). The indices of simulation efficiency including relative error (*RE*), linear regression coefficient (*R* ^2^) and Nash–Sutcliffe simulation efficiency coefficient (*E*_*ns*_) were used to evaluate the simulation results. The calculation methods of *RE*, *R* ^2^ and *E*_*ns*_ are shown as follows:

Relative error (*RE*)

$$RE=\frac{E_{s,i}-E_{m,i}}{E_{m,i}}$$ (17)

where *E*_*s*,*i*_ is the simulated value of summer maize evapotranspiration, *E*_*m*,*i*_ is the measured value of summer maize evapotranspiration.

Linear regression coefficient (*R* ^2^)

$$R^{2}=\frac{{\left[\sum_{i=1}^{n}\left(E_{m,i}-\bar{E_{m}}\right)\left(E_{s,i}-\bar{E_{s}}\right)\right]}^{2}}{\sum_{i=1}^{n}{\left(E_{m,i}-\bar{E_{m}}\right)}^{2}\sum_{i=1}^{n}{\left(E_{s,i}-\bar{E_{s}}\right)}^{2}}$$ (18)

where $\bar{E_{s}}$ is the average simulated value of summer maize evapotranspiration, $\bar{E_{m}}$ is the average measured value of summer maize evapotranspiration.

Nash–Sutcliffe simulation efficiency coefficient (*E*_*ns*_)

$$E_{ns}=1-\frac{\sum_{i=1}^{n}{\left(E_{s,i}-E_{m,i}\right)}^{2}}{\sum_{i=1}^{n}{\left(E_{m,i}-\bar{E_{m}}\right)}^{2}}$$ (19)

where *E*_*ns*_ ranges from 0 to 1, when *E*_*ns*_ is close to 1, the simulated value is close to measured value, and vice versa.

According to the evaluation requirements of the simulation results in SWAT, the relative error of annual evapotranspiration should be less than ±20%, the linear regression coefficient and Nash–Sutcliffe simulation efficiency coefficient of monthly evapotranspiration should be higher than 0.6 and 0.5, respectively.

### 3.6 Planting scenarios

In order to reduce the agricultural water consumption, the policy of cutting down the planting proportion of grain crop of high water consumption and increasing the corresponding cultivated area of economic crops have been provided by the local government in Qingyuan Irrigation District. The crop evapotranspiration response to the current situation and two different planting scenarios were employed in the irrigation district. For each scenario, the output of different crops must meet the living condition of the local residents (i.e. for the production of grain, oil or sugar). Scenario 1 assumed that summer maize planting proportion kept unchanged, the planting proportion of high water consumption crop of spring wheat would be reduced to 20% and other economic crop scenarios would be adjusted according to local policies under the condition of total cultivated area remaining unchanged (Table 6). Scenario 2 assumed that spring wheat planting proportion kept unchanged, the planting proportion of high water consumption crop of summer maize would be reduced to 9.5% and other economic crop scenarios would be adjusted according to local policies under the condition of total cultivated area remaining unchanged (Table 6).

**Table 6:** Different planting scenarios in Qingyuan Irrigation District.

Crop	Current Situation (%)	Scenario 1 (%)	Scenario 2 (%)
Spring wheat	40	20	40
Summer maize	30	30	9.5
Potato	12	5	16.8
Soybean	3	10	5
Beet	5	5	5
Chili	10	10	13.8
Watermelon	0	20	9.9

## Results and Discussion

### 4.1 Sensitivity analysis

The results of sensitivity analysis are shown in Table 5. The parameter ranked 1st was considered the most important, while parameter ranked 5th was the least important. The sensitivity results were ranked according to the descending order of relative importance as soil evaporation compensation factor, maximum canopy interception, shallow groundwater re-evaporation coefficient, plant absorption compensation factor and soil available water. The parameter representing the process of evaporation was the most important. Thus, accurate estimation of these parameters is important for evapotranspiration simulation in the irrigation district.

### 4.2 Model calibration and verification

**Fig 8:**
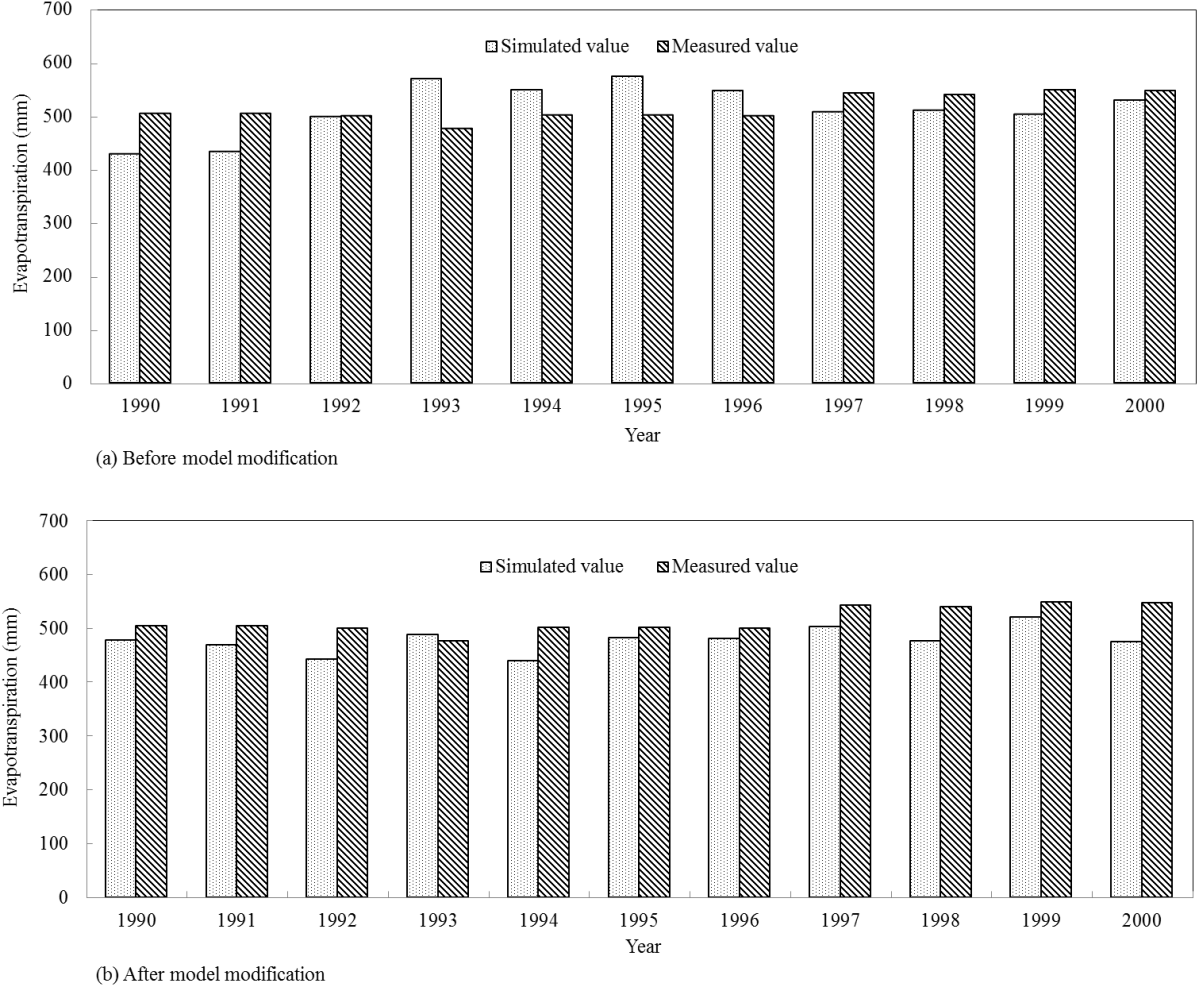
Simulated and measured values of summer maize annual evapotranspiration in the calibration period.

**Fig 9:**
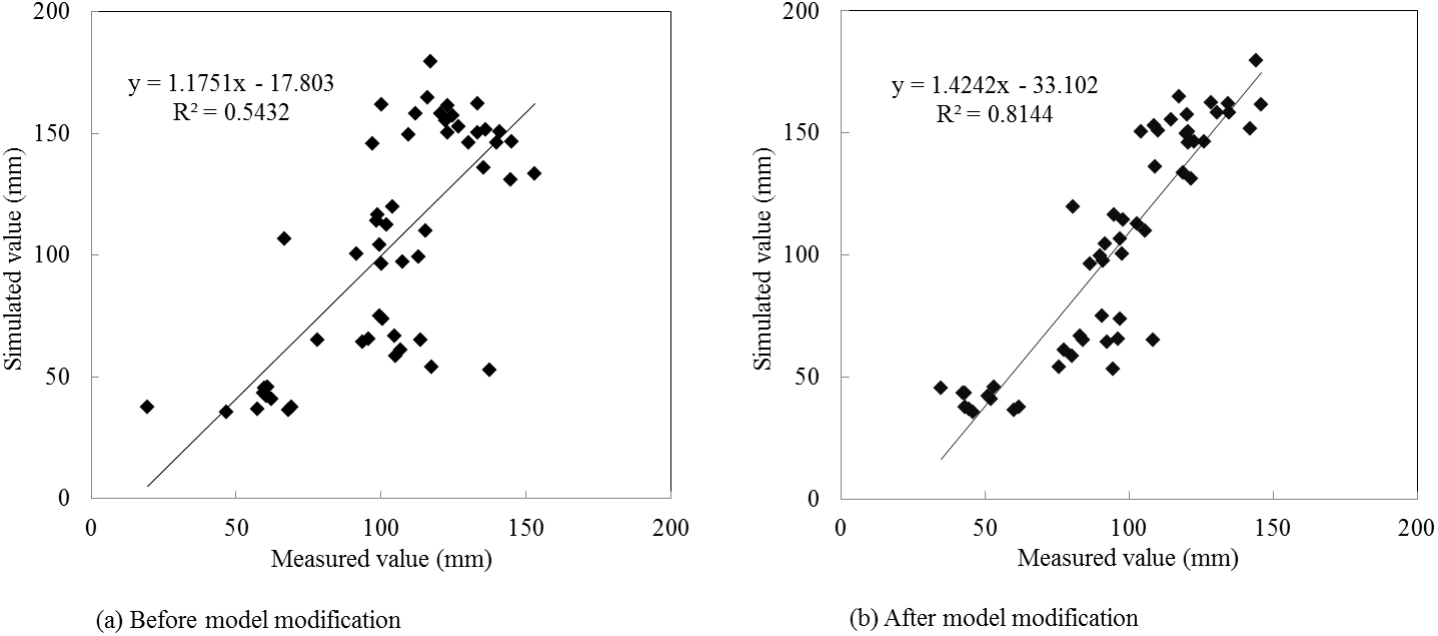
Simulated and measured values scatter diagram of summer maize monthly evapotranspiration in the calibration period.

The simulation results in the calibration period are presented as Table 7, and Figs 8 and 9. During the calibration period (1990–2000), the *RE* values of summer maize annual evapotranspiration ranged from -14.80% to 19.78% before model modification. By contrast, the *RE* values were from -13.24% to 2.67% after improving the evapotranspiration module, which presented a better simulation result compared with the original model. Before model modification, the *R* ^2^ and *E*_*ns*_ values of summer maize monthly evapotranspiration were 0.54 and 0.53, while the *R* ^2^ and *E*_*ns*_ values were 0.81 and 0.72 after model modification with the figure increasing by 50.00% and 35.85%, respectively, compared with the original model. In the calibration period, both the original and modified model versions met the evaluation requirements of the SWAT model, but the modified model showed higher simulation efficiency.

**Table 7:** Simulation efficiency of original and modified model versions in the calibration and verification periods.

calibration period			verification period		
Year	Simulation efficiency		Year	Simulation efficiency	
	Original model	Modified model		Original model	Modified model
	*RE* (%)			*RE* (%)	
1990	-14.80	-5.21	2006	-9.57	-1.79
1991	-14.26	-7.06	2007	-16.00	-6.26
1992	-0.15	-11.52	2008	-37.55	-9.57
1993	19.78	2.67	2009	-31.47	-9.74
1994	9.46	-12.44	2010	-22.32	-11.83
1995	14.55	-3.65	2011	-12.09	-1.87
1996	9.62	-3.69			
1997	-6.54	-7.39			
1998	-5.47	-11.85			
1999	-8.26	-5.25			
2000	-3.13	-13.24			
1990–2000	*R* ^*2*^		2006–2011	*R* ^*2*^	
	0.54	0.81		0.75	0.88
1990–2000	*E*_*ns*_		2006–2011	*E*_*ns*_	
	0.53	0.72		0.45	0.71

**Fig 10:**
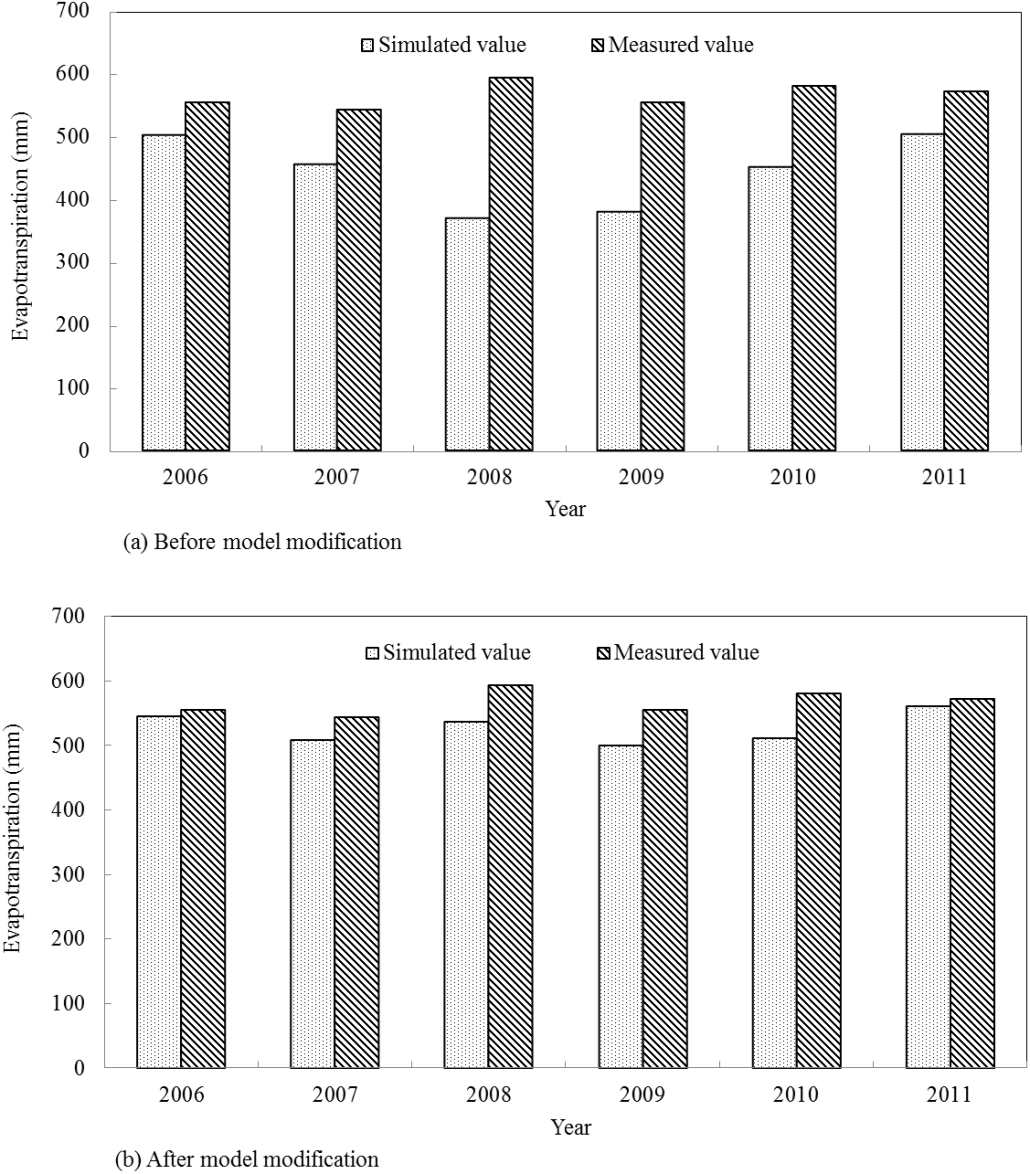
Simulated and measured values of summer maize annual evapotranspiration in the verification period.

**Fig 11:**
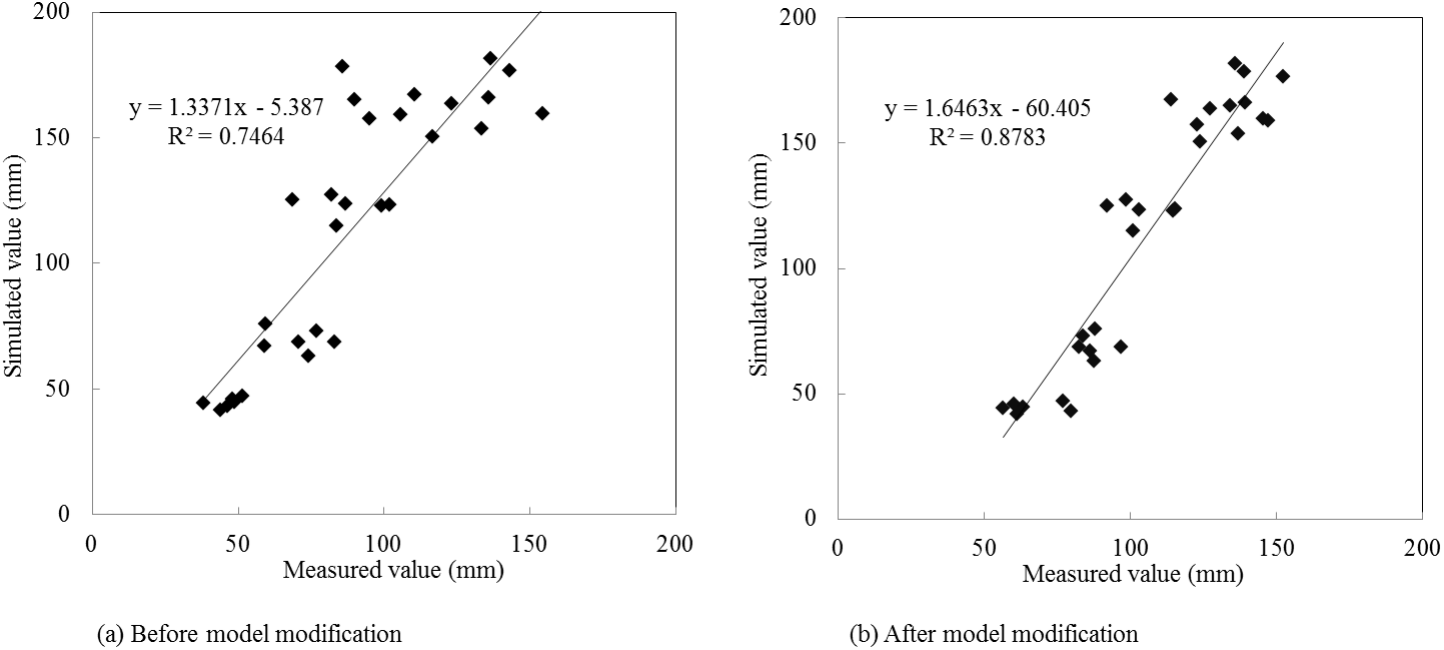
Simulated and measured values scatter diagram of summer maize monthly evapotranspiration in the verification period.

The simulation results in the verification period are presented as Table 7, and Figs 10 and 11. During the verification period (2006–2011), the *RE* values of summer maize annual evapotranspiration varied from -37.55% to -9.57% before model modification, while the *RE* values were between -11.83% and 1.79% after improving the evapotranspiration module. The relative errors which were relatively large in the year 2008, 2009 and 2010 decreased by 74.51%, 69.05% and 47.00%, respectively, after model modification. The *R* ^2^ and *E*_*ns*_ values of summer maize monthly evapotranspiration were 0.75 and 0.45 in the original model. By contrast, the *R* ^2^ and *E*_*ns*_ values were 0.88 and 0.71 in the modified model, which increased by 17.33% and 57.78%, respectively. In the verification period, only the modified model met the evaluation requirements of the SWAT model and showed higher simulation efficiency. Therefore, the modified model is more suitable for evapotranspiration simulation in Qingyuan Irrigation District of arid area of northwest China.

### 4.3 Crop evapotranspiration response to different planting scenarios

**Fig 12:**
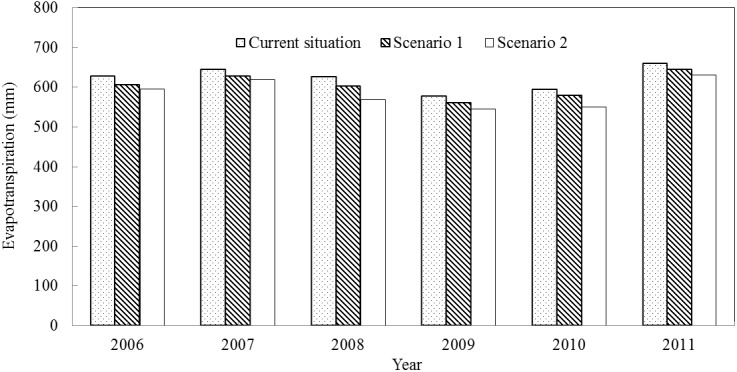
Crop evapotranspiration responses to different planting scenarios.

The modified SWAT model was used to simulate the crop annual evapotranspiration in Qingyuan Irrigation District under two different planting scenarios for the period from 2006 to 2011. As can be seen from Fig 12, the changing trends of crop evapotranspiration from 2006 to 2011 were similar under current scenario, scenario 1 and scenario 2. Besides, the crop evapotranspiration under scenario1 and scenario 2 were less that of current scenario for different years. The average annual crop evapotranspiration under current situation was 622.70 mm. The average annual evapotranspiration under scenario 1 and scenario 2 were 604.38 mm and 585.24 mm, which decreased by 2.94% and 6.01%, respectively, compared with current situation. The reason for the decrease of evapotranspiration under scenario 1 and scenario 2 were mainly because the decrease of cultivated areas of high water consumption crop of spring wheat and summer maize.

The crop output values under current situation and different scenarios are shown in Table 8. The total net output value under scenario 1 was 138.31×10^6^ yuan, which decreased by 3.28% compared with current situation. However, the total net output value under scenario 2 increased by 7.79% with the value of 154.13×10^6^ yuan. The main reason was because the unit net output value of potato was the highest (15688yuan/hm^2^), while the unit net output value of soybean is the lowest (1320yuan/hm^2^). In scenario 1, the planting areas of potato decreased from 12% to 5% of the total cultivated areas, and the planting areas of soybean increase from 3% to 10%, which caused the decrease of total net output value. In scenario 2, the planting areas of potato increased from 12% to 16.8% of the total cultivated areas, which cause the increase of total net output value.

**Table 8:** Crop output values in Qingyuan Irrigation District.

Crop	Yield (kg/hm^2^)	Unit price (yuan/kg)	Unit input value (yuan/hm^2^)	Unit output value (yuan/hm^2^)	Cultivated area (hm^2^)			Total net output value (10^6^ yuan)		
					Current Situation	Scenario 1	Scenario 2	Current Situation	Scenario 1	Scenario 2
Spring wheat	6510	2.1	9525	4146	9357.2	4678.6	9357.2	38.79	19.40	38.79
Summer maize	9900	2.0	13610	6190	7017.9	7017.9	2222.3	43.44	43.44	13.76
Potato	33110	0.8	10800	15688	2807.2	1169.7	3930.0	44.04	18.35	61.65
Soybean	2100	3.2	5400	1320	701.8	2339.3	1169.7	0.93	3.09	1.54
Beet	31500	0.5	10500	5250	1169.7	1169.7	1169.7	6.14	6.14	6.14
Chili	18750	1.1	16500	4125	2339.3	2339.3	3228.3	9.65	9.65	13.32
Watermelon	34872	0.6	12750	8173.2	0	4678.6	2315.9	0	38.24	18.93
Total	-	-	-	-	23393.1	23393.1	23393.1	142.99	138.31	154.13

The evapotranspiration showed a downward trend under scenario 1 and scenario 2 when compared with current situation. In terms of total net output values, scenario 1 presented a- downward trend, while scenario 2 showed an opposite trend. Thus, taking all factors into account, planting under scenario 2 was more suitable for the development of Qingyuan Irrigation District. Besides, according to the research result, the local residents can also increase the planting areas of crops with low water consumption and high unit output value (i.e. potato and watermelon), and decrease the planting areas of crops with high water consumption or low unit output value (i.e. spring wheat and soybean).

## Conclusions

The SWAT model incorporating the improved evapotranspiration module (FAO–56 dual crop coefficient method) was developed for studying the hydrological processes in an irrigation district of evapotranspiration-dominant arid area. Then, the original and improved models were used for model calibration and verification. As the improved model showed better simulation efficiency, it was applied to simulate crop evapotranspiration response to different planting scenarios in Qingyuan Irrigation District of northwest China.

The results showed that crop evapotranspiration decreased by reducing the planting proportion of high water consumption crops (spring wheat and summer maize) and adjusting the proportion of economic crop cultivated areas under the condition of total cultivated area remaining unchanged. However, the total net output values presented an opposite trend by cutting down the planting proportion of spring wheat (upward) and summer maize (downward) compared with the current situation.

In general, the study results can be used to evaluate the impact of planting scenario change on crop evapotranspiration in the irrigation district of arid area of northwest China, which plays a significant role in optimizing crop planting, resolving the water resource crises and reducing ecological deterioration. The results provide recommendations for strategic water management and planning in the irrigation district.

## Supporting Information

S1 File Granted permission. (DOCX)

S2 File Original GIS data. (RAR)

S3 File Original data from Fig 8 to Fig 12. (XLSX)
