# Supplementary material for: Simulating Crop Evapotranspiration Response under Different Planting Scenarios by Modified SWAT Model in an Irrigation District, Northwest China
Source: PLoS One. 2015 Oct 6;10(10):e0139839. doi: 10.1371/journal.pone.0139839 (PMC4595335; doi:10.1371/journal.pone.0139839)
Supplement: S1 File — (DOCX) [file pone.0139839.s001.docx]

**Granted permission**

I request permission for the open-access journal PLOS ONE to publish under the Creative Commons Attribution License (CCAL) CC BY 4.0.

Figures 2-7 are reprinted from *Impacts of Agricultural Management Practices on Hydrological Cycle in Qingyuan Irrigation Districts* under a CC BY license, with permission from China Agricultural University, original copyright 2013.
